# Aging Effects on Phonological and Semantic Priming in the Tip-of-the-Tongue: Evidence From a Two-Step Approach

**DOI:** 10.3389/fpsyg.2020.00338

**Published:** 2020-02-27

**Authors:** Mingkun Ouyang, Xiao Cai, Qingfang Zhang

**Affiliations:** Department of Psychology, Renmin University of China, Beijing, China

**Keywords:** tip-of-the-tongue, transmission deficit hypothesis, two-step approach, phonological priming, semantic priming, aging

## Abstract

The mechanism underlying the age difference in spoken word production remains controversial. We used a two-step approach proposed by Gollan and Brown (2006) to investigate the semantic and phonological retrieval deficits when tip-of-the-tongue occurs in young and older adults. Importantly, we controlled the inhibition ability in both older and young groups. In experiment 1 with a people pictures naming task, older adults produced more TOTs than young adults, and they suffered from phonological retrieval deficit rather than semantic retrieval deficit in speaking. In experiment 2 with a priming paradigm, participants were presented semantically related or phonologically related names before target pictures, which formed semantic or phonological priming conditions for lexical access. Compared with young adults, older adults showed a greater effect of phonological priming on decreasing TOTs occurrence. For semantic retrieval deficit, older adults exhibited a smaller phonological facilitation effect and a larger semantic interference effect than young adults. For phonological retrieval deficit, older adults presented a larger phonological facilitation effect in the first-name related priming condition than the first-syllable related priming condition, whereas young adults showed similar facilitation effects between the two phonological priming conditions. Our findings provide consistent evidence for the transmission deficit hypothesis, and highlight that aging affects bidirectional connections between semantic and phonological nodes in speech production.

## Introduction

Spoken production involves a sequence of planning stages, including conceptual preparation, lexical selection, word-form encoding and articulation (Levelt et al., 1999). Aging of spoken production gains its attention since retrieval failures become more prevalent as people age (Mortensen et al., 2006; Shafto and Tyler, 2014). Older speakers frequently report more linguistic dysfluencies, verbose and even anomia in speech production than young speakers (Cavanaugh et al., 1983; Cohen and Faulkner, 1986). Indeed, word-finding failures are considered as the most frequent, annoying and embarrassing cognitive failure for older adults (Sunderland et al., 1986; Lovelace and Twohig, 1990).

Tip-of-the-tongue (henceforth TOT) is a typical word-finding failure which presents that a person cannot access a known word from memory although one feels the missing word is about to be retrieved. Converging evidence from diary studies of naturally occurring TOTs (Burke et al., 1991; Heine et al., 1999) and laboratory studies (Rastle and Burke, 1996; James and Burke, 2000; Cross and Burke, 2004; Farrell, 2012; Salthouse and Mandell, 2013) shows that TOT increases with normal aging. TOT occurs nearly every day for older adults but once a week for young adults (Brown, 2012). Most accounts attribute it to either specialized information retrievals and/or transmission failure, or general cognition impairments. The present study aims to elucidate the mechanism underlying differences in TOT occurrence between young and older adults.

### Asymmetries of Semantic and Phonological Retrieval in Old Age

Studies reported that semantic processing remains stable in old age in language comprehension and production (Kavé and Mashal, 2012; Ramscar et al., 2014; Lacombe et al., 2015) since semantic process of language benefits from verbal knowledge (i.e., vocabulary and semantic memory) adults have acquired over years (Verhaeghen, 2003; Ben-David et al., 2015). Compared to young adults, older adults presented comparable performance in semantic processing tasks including semantic judgment (Little et al., 2004), semantic priming task (Gold et al., 2009), lexical decision task (Gold et al., 2009), and picture naming (Kavé and Mashal, 2012). Farrell (2012) compared semantic priming of TOTs occurrence for word production in young and older adults, and did not find the effect of semantic priming on TOTs for either age group, reflecting that TOTs are little associated with word’s semantics (see also Cross and Burke, 2004). By contrast, studies demonstrated an aging effect on phonological retrieval in spoken production. Using a phonological priming task, Oberle and James (2013) reported that older speakers benefited more from phonological primes than young speakers in solving TOTs, which suggests that there is a weaker connection between semantics and phonology for older speakers.

However, findings regarding the aging of phonological encoding were not consistent. James and Burke (2000) used a phonological priming task in which phonologically related words were presented before participants retrieving target words, and observed that there was no age difference in phonological priming effect in TOTs occurrence. Similar results were also found in TOTs resolution, for instances, adults in their 1960s and early 1970s showed comparable phonological priming of TOTs resolution as young adults (White and Abrams, 2002; Abrams et al., 2007).

There are two possibilities to explain these discrepant findings. Firstly, given that the age of older participants varied across studies, the degree of age-related decline in transmission deficit may also vary. People in their late 1970s and 1980s exhibited an exacerbation of transmission deficit, which resulted in less or no phonological priming in TOTs resolution in comparison with young and young-old adults (1960–1970s) (Heine et al., 1999; White and Abrams, 2002; Abrams et al., 2007). Secondly, the rates of TOTs measured in these studies may be insensitive to the priming manipulation since the rates of TOTs induced in most studies were generally very low. Thus, it is necessary to use a sensitive approach to examine TOTs occurrence in spoken word production.

### Information-Specific and Information-Universal Accounts for Aging Effect in Spoken Word Production

**FIGURE 1:**
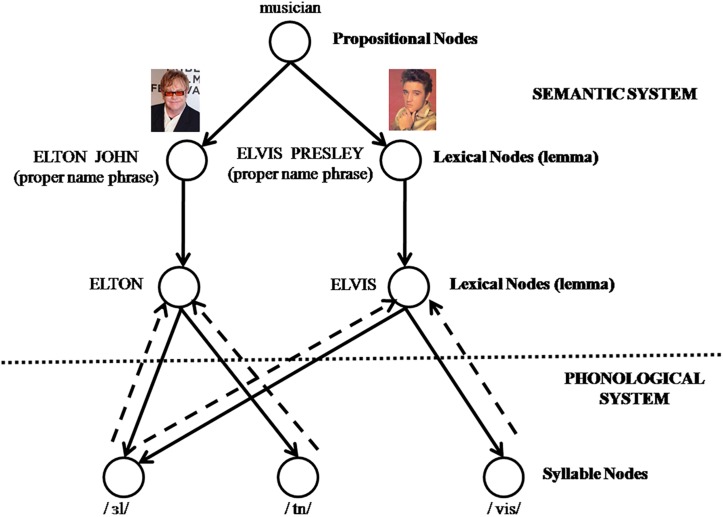
A simplified illustration of how activation transmits between lexical nodes and phonological nodes. When a lexical node (e.g., Elton) becomes activated in the lexico-semantic level, semantic activation will spread and transmit top-down priming to its corresponding phonological nodes (e.g., /_³_l/and/tn/) via top-down connections represented by solid lines. As a result, phonological nodes would be activated to some extent. According to the interactive activation models, activation can feedback from phonological nodes (e.g., /_³_l/) to lemma nodes (e.g., Elton and Elvis) via bottom-up connections represented by dotted lines.

To explain the aging effects in speech production, Burke and colleagues proposed a *transmission deficit hypothesis* (henceforth TDH) in the information-specific framework (MacKay and Burke, 1990; Burke et al., 1991; Burke and Shafto, 2004). The TDH assumes that aging weakens the connection between semantic and phonological representations, which results in a reduction in the transmission of semantic priming and then makes activation of phonological representations more difficult (see Figure 1). Therefore, compared with young speakers, older speakers generally report more TOTs. Empirical findings provided evidence for this hypothesis by showing a dissociation between semantic and phonological retrievals during TOTs occurrence (Thornton and Light, 2006; Burke and Shafto, 2008). Speakers can correctly retrieve lexical-syntactic properties of target words such as countability (count or mass), grammatical gender (masculine or feminine) and number (singular or plural), while they cannot retrieve complete sound forms (i.e., phonological segments) (Vigliocco et al., 1999; Schwartz, 2002). In a proper name production (i.e., person’s name) study, Cohen and Faulkner (1986) reported that people in a TOT state could retrieve semantic information about target persons such as their occupation or hobbies, but hardly speak out the names. In addition, previous studies using a priming procedure in which a priming word was presented before or after TOTs occurrence found that semantic primes did not reduce TOTs occurrence (Farrell, 2012) or improve TOTs resolution (Cross and Burke, 2004), whereas phonological primes played a pivotal role in reducing TOTs occurrence (Abrams and Rodriguez, 2005; Farrell and Abrams, 2011; Pureza et al., 2013) and enhancing TOTs resolution (Oberle and James, 2013; Pureza et al., 2013; White et al., 2013).

Of special interest for the present research is the study of Juncos-Rabadán et al. (2010), which examined the semantic and phonological retrievals of proper names when TOTs occurred in speakers aged from 19 to 82 years, using a two-step approach (see section “The Two-Step Approach” for details). In the experiment, participants were presented with definitions and were asked to produce names corresponding to definitions. Participants’ responses were divided into 3 types: I know it (GOT), I don’t know it (DK), and I know it but can’t say it out (TOT). TOTs were further divided into positive (pTOT) and negative TOT (negTOT) based on whether participants can recognize the target names or not. Juncos-Rabadán et al. focused on the success in semantic retrieval and the failure in phonological retrieval in TOTs occurrence, which were assessed by equations of (GOTs + pTOTs + negTOTs)/N and pTOTs/(pTOTs + GOTs), respectively. Juncos-Rabadán and colleagues found that older speakers produced more proportion of pTOTs, and obtained higher scores for success in semantic retrieval and for failure in phonological retrieval than young speakers. Besides, the age difference between young speakers (i.e., 19–26 years) and older speakers (i.e., 70–82 years) in phonological retrieval failure was still significant after matching vocabulary size, but age difference in semantic retrieval became non-significant. Juncos-Rabadán et al. thus suggested that older speakers show greater semantic retrieval efficiency while more impaired phonological access than young ones. Based on the literature reviewed above, it is plausible to assume that age-related increase in TOTs may arise from a difficulty in phonological retrieval rather than in semantic retrieval (Levelt et al., 1999; Gollan and Brown, 2006). So far, it remains unknown whether the age-related phonological impairment increases due to transmission deficit between semantics and phonology (Mirman and Britt, 2014), insufficient activation of phonological representations (Brown and McNeill, 1966), or inhibition deficit of distractors (Jones and Langford, 1987; Jones, 1989).

From a different perspective, information-universal theories assume that domain-general cognition such as processing speed, working memory, executive functions (Gunning-Dixon and Raz, 2003; Park and Reuter-Lorenz, 2009; Ebaid et al., 2017) and inhibitory processes (Hasher and Zacks, 1988; Hasher et al., 2007) decline with aging. Many empirical studies have reported that non-specific cognitive decline resulted in not only increased latencies in picture naming and semantic processing (Shao et al., 2012; Boudiaf et al., 2018), but also highly dysfluent speech (Mortensen et al., 2006; Engelhardt et al., 2013; Korko and Williams, 2017). According to the *inhibition deficit hypothesis* (henceforth IDH) (Jones and Langford, 1987; Jones, 1989), normal aging disrupts inhibitory mechanisms, rendering reduced ability to suppress irrelevant or competing information (Hasher and Zacks, 1988; Hasher et al., 2007). This age-related decline has been observed in a variety of tasks such as attention processing (Bunce et al., 1993; Stoltzfus et al., 1993), and episodic memory recall (Hartman and Hasher, 1991). Therefore, it is necessary to control the general inhibition ability between young and older speakers when investigating spoken word production in the framework of information-specific accounts.

### The Two-Step Approach

Spoken word production involves two basic retrieval stages, that is, meaning-based (step 1: lexical selection) and form-based retrieval (step 2: word-form encoding) (Dell, 1986; Roelofs, 1997; Levelt et al., 1999). Gollan and Brown (2006) proposed a two-step approach to estimate the probability of semantic and phonological retrieval failures when TOTs occur. In this method, there are five types of responses with reference to success versus failure in the two steps of lexical access in speaking. A GOT (Got the words) reflects a successful retrieval of semantics and phonology for both step 1 and step 2. A positive TOT reflects a successful retrieval of semantics (step 1) but a failed retrieval of target’s phonology (step 2), while a negative TOT reflects a failed retrieval of both semantics (step 1) and phonology (step 2) for intended target word. A notGOT response refers to a state in which speakers may recognize the correct target word, even though they fail to retrieve it in their first attempt. Speakers may report that they do not know the target word (don’t know, DK response) or cannot recognize the intended word after being presented with it (post-don’t know, post-DK response). Positive TOTs and GOTs reflect successful retrieval of semantic information while positive TOTs reflect the responses that only completed step 1 but failed step 2. Gollan and Brown (2006) suggested the below equation to calculate probability of phonological retrieval deficiency.

$$\frac{p\,o\,s\,i\,t\,i\,v\,e\,T\,O\,T\,s}{G\,O\,T\,s+p\,o\,s\,i\,t\,i\,v\,e\,T\,O\,T\,s}$$

All other responses (negative TOT, DKs, and postDKs) reflect a failure in step 1. Thus, probability of semantic retrieval deficiency is estimated using the below equation (N is the total number of target words).

$$\frac{N-\left(G\,O\,T\,s+T\,O\,T\,s\right)}{N}$$

Using the two-step approach, Gollan and Brown (2006) revealed that aging has greater negative effects on phonological retrieval than semantic retrieval for (difficult) target words. By this well-established approach, previous studies found that word concreteness, word frequency (Gianico-Relyea and Altarriba, 2012), mild cognitive impairment (Gollan et al., 2014), and word length (Hanley and Chapman, 2008) influenced phonological retrieval alone, while target familiarity influenced both semantic and phonological retrieval (Hanley and Chapman, 2008), suggesting that this approach is sensitive to semantic and phonological retrievals in spoken word production.

### The Current Study

So far, rare studies have exploited this approach to investigate aging effect on proper name retrieval during TOTs (Gollan and Brown, 2006; Juncos-Rabadán et al., 2010). Compared with common names, proper names (e.g., people names) are vulnerable to retrieval deficit because their functional architecture, in particular, the absence of multiple semantic connections, makes them vulnerable to transmission deficit at both lexical-semantic and phonological levels (Cohen, 1990; Evrard, 2002; Semenza, 2006; Fogler et al., 2010; Abrams and Davis, 2017). In other words, production of proper names may be subject to semantic retrieval deficit in addition to phonological retrieval deficit. The lack of age-related semantic retrieval deficit may be partially because related alternatives co-activated during proper name retrieval are not too many and then can be successfully suppressed by older adults to prevent interference with target’s semantic retrieval. Hanley (2011) compared the TOTs occurrence between proper names and common names, and reported more TOTs and phonological retrieval failures during attempts to retrieve proper names than common names. Importantly, when the number of alternatives was equated when participants produced proper or common names, there were no differences in the TOTs occurrence, semantic and phonological retrievals. This finding indicated that the aging effect on proper name retrieval during TOTs occurrence may be associated with general inhibition ability, which could further modulate aging effect on semantic and phonological retrievals. Thus, the current study firstly aims to investigate how aging affects proper name retrieval (i.e., semantic and phonological retrievals) using a two-step approach when age-related differences in general inhibition ability were controlled (Engelhardt et al., 2013).

In the TDH’s framework, there are bidirectional activation in the connection between semantic and phonological representations in speaking (see Figure 1). In a picture naming task with words as primes, the perception of prime words activates phonological nodes that transmit bottom-up priming, which is less likely to result in a transmission deficit at the lexical level because of the activation from phonological nodes to a lexical node. While the production of target words activates lexical nodes that transmit top-down priming from semantics to phonology, in which priming diverges from a lexical node to corresponding phonological nodes via single connection. The TDH thus predicts an age-related increase in TOTs due to the weakened connections from semantics to phonology via top-down priming of phonological information, and predicts no aging effect in bottom-up phonological priming or in semantic priming.

So far, whether aging affects bottom-up phonological priming or semantic priming remains unclear. It is possible that this process might be age-sensitive, and was inevitably subjected to transmission failure. Empirical evidences have demonstrated that normal aging weakens not only top-down activation from semantics to phonology, but also bottom-up activation from phonology to semantics (Robert and Mathey, 2007; Robert and Duarte, 2016). Gordon and Kurczek (2014) reported that the effect of phonological neighborhood density on word production was facilitative in young adults but inhibitive in older adults, which suggests that there is an age-related decline in the activation transmission from shared phonemes to related lexical nodes (i.e., semantics). TOTs are usually accompanied with persistent alternatives (Burke et al., 1991; Heine et al., 1999; Kornell and Metcalfe, 2006), but the number of alternatives tends to be fewer for older adults because the bottom-up priming to the lexical nodes of alternates is decreased (Burke et al., 1991). Some studies even showed that older adults frequently experienced an “empty gap,” with less partial phonological information about target words and fewer competitor words retrieved in TOTs (Cohen and Faulkner, 1986; Burke et al., 1991; Brown and Nix, 1996; Heine et al., 1999).

Recent findings in Mandarin Chinese revealed that the relation between semantic and phonological processes is serial and discrete in spoken word production for young native speakers (Zhu et al., 2015, 2016; Zhang et al., 2018). That is, there is a unidirectional transmission from semantic nodes to phonological nodes in Chinese spoken word production. However, we should remain cautious when generalize this conclusion to older adults. Therefore, the present study aims to examine whether there is an age-related deficit for bottom-up phonological and semantic priming.

Tip-of-the-tongues studies on people name retrieval usually involve face processing as well. The face-naming models assume that semantic and phonological retrievals occur in parallel, that is, activations from person identity nodes (PINs) are transmitted to identity-specific semantic and phonological nodes simultaneously, and there are bi-directional links between PINs and semantic or phonological nodes (Burton et al., 1990; Valentine et al., 1991, 1996). The PINs functioning as token markers provide access to person semantic information (e.g., nationality, occupation, and marital status) or name information, that is, to lexical and phonological nodes. Based on the bidirectional links between PINs and lexicons, semantic and phonological primes would help activating the PINs, and reduce the occurrence of TOTs. Semantic and phonological primes may exert their influences by different mechanisms, and we will discuss them in the general discussion.

In experiment 1 we use a people pictures naming task to examine whether age-related increase in TOTs is associated with semantic or phonological retrieval deficit. In experiment 2, we use a priming task to examine whether there is an age-related deficit in bottom-up phonological and semantic priming by manipulating semantically related or phonologically related words with target picture names. The two-step approach and equations proposed by Gollan and Brown (2006) were used in both experiments. Importantly, we exclude the potential influence of the general inhibitory ability measured by a Stroop color-word task and functioned as a covariate in the mixed ANCOVAs analysis. Under the TDH, we predict that age-related increase would present more failures in phonological rather than semantic retrievals in experiment 1. Furthermore, for the probability of semantic retrieval deficit, we predict that phonologically related priming would facilitate semantic retrieval via bottom-up priming, and this priming effect would be mediated by aging in experiment 2. Especially, considering that the bottom-up pathways from phonology to semantics may be sensitive to aging, older adults would benefit less from the phonological priming for decreasing the semantic retrieval deficit during TOTs occurrence than young adults. In addition, we predict that, compared with young adults, older adults will produce a disproportionally phonological facilitation effect, for the reduction of TOTs occurrence and phonological retrieval deficit, in different phonological priming conditions. Given that the forward activation from semantic to phonological representation is particularly susceptible to transmission failure as people age, the facilitation effect could be more evident under strong priming condition.

## Experiment 1

### Methods

#### Participants

Participants included 40 young (18–28 years; *M* = 20.63, *SD* = 2.35) and 42 older native Mandarin speakers (60–72 years; *M* = 65.78, *SD* = 6.89). Young adults were recruited from Renmin University of China, and older adults from a local community in Beijing. All reported normal or corrected to normal vision, and none of them had a history of language impairment, psychiatric disorders or neurological disease. The two age groups did not differ in years of education (*F* (1, 70) = 0.814, *p* = 0.37, η_*p*_^2^ = 0.011). Older adults were additionally screened for cognitive deficit using the Montreal Cognitive Assessment Scale (MoCA; Nasreddine et al., 2005). Three older participants were removed because of their low scores on the MoCA. Scores for the rest of the participants were above a cut-off of 26 (*M* = 27.51, *SD* = 1.33). All participants were given written informed consent and were paid approximately $12 for their participation. The study was approved by the Renmin University Research Ethics Committee.

#### Materials

Stimuli were photographs of the faces of famous people from the following occupations: actor, or actress; TV host; scholar; singer; sportsman; politician; businessman. The photographs were obtained from different sources including television, newspaper, magazines and the internet. Then twelve young and twelve older adults named 200 photographs and rated their familiarity on a five-point Likert scale (1 = *very low*; 5 = *very high*) in a pilot study. We finally chose 109 familiar faces as target stimuli in our study. The familiarity for young participants (*M* = 3.18 ± 0.28) and older participants (*M* = 3.30 ± 0.23) was matched, *t* (22) = –1.145, *p* = 0.264. The materials used were presented in Supplementary Appendix A.

#### Apparatus

The experiment was performed using E-Prime Professional Software 2.0. The pictures were presented on a high-resolution monitor of 1024 × 768. The response was recorded by a microphone, which connected with the Lenovo-compatible PC via a PST Serial Response Box.

#### Procedure and Scoring

Our paradigm was adapted from Gollan and Brown (2006) with an adjustment that we used familiar faces of celebrities instead of object pictures. Participants were tested individually. The experiment began with a practice block of 10 trials to familiarize participants with the procedure. After that, participants finished 109 randomly presented experimental trials.

**FIGURE 2:**
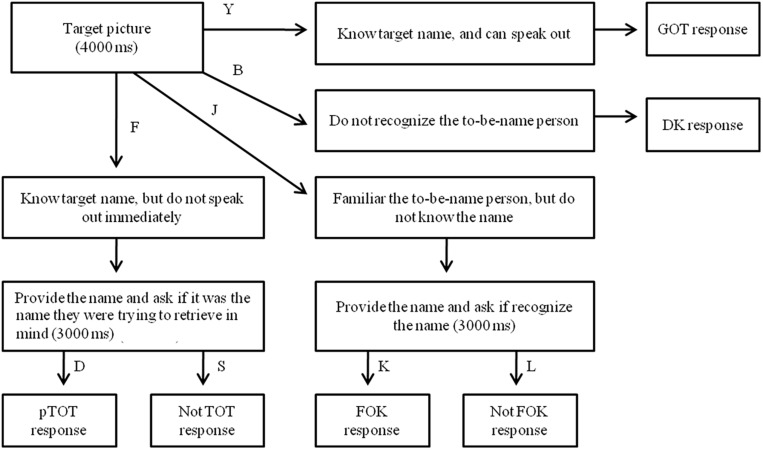
Procedure order and scoring categories for each response type in Experiment 1. GOT, Correct retrieval of target name; pTOT, Positive tip-of-the-tongue; Not TOT, Failure to retrieve a known word; DK, Don’t know the target name; FOK, Feeling of knowing the target person; Not FOK, Familiar with the target person but do not know the name.

Prior to the experiment, all participants were told that a TOT is “when you are sure you know a name but can’t remember it at that moment” (Gollan and Brown, 2006; Gollan et al., 2014). Participants were tested individually. Each trial began with a 500 ms presentation of a small black cross on the white computer screen. Then a photograph was presented for 4000 ms, and participants were required to respond with a key press within 4000 ms from stimulus onset. The button response was classified into four major types depending on whether they knew or recognized the name of the celebrity: (1) If they know the name and can produce at once, then they press the button “Y” and correctly say the person’s name aloud (GOT response, as in “got it”; Gollan and Brown, 2006; Gollan et al., 2014); (2) If they surely know the name, but cannot produce it immediately, they press the button “F” with the left index. After the press, the target name will be presented for 3000 ms in the middle of the screen. Participants have to decide whether the presented name was the name they were trying to retrieve by pressing buttons, with “D” representing the to-be-produced name (positive TOT response, pTOT) and “S” representing not the to-be-produced name (negative TOT response or not TOT response, nTOT) (Gollan and Brown, 2006); (3) If they are familiar with the person but surely do not know the name, or know the person’s name but the name is not on the verge of producing, they have to press the button “J” (feeling of knowing, FOK). After the press, the target name will be presented for 3000 ms and participants were asked if recognize the name by pressing buttons, with “K” representing the name correctly recognized and “L” representing the name incorrectly recognized; (4) If they are sure that they do not know the person at all, press the button “B” (don’t know response, DK). The presentation of photograph was terminated by a key pressing, or was terminated when the photograph elapsed for 4000 ms (Figure 2). A 3-min break was used after 60 trials to avoid the fatigue effect.

To assess general inhibitory ability, all participants completed a Stroop color-word Task. In this task, stimuli consisted of Chinese color word names red, yellow and green displayed in Arial fonts with the ink colors red, yellow or green. Participants had to indicate the ink color of the stimulus as fast as possible by pressing the buttons “1,” “2,” or “3” on the keyboard using the middle, index or ring finger of their right hand. Stimuli were randomly presented in the center of the screen and disappeared until a button response. There were 90 trials in total for nine possible word-color combinations, including 30 congruent trials and 60 incongruent trials.

### Results

Four young and three older participants were excluded from the analysis because they reported no TOT occurrence in the experiment. Data from incorrect responses (2.01% and 1.38% for young and older participants) were removed from subsequent analyses. For each participant, we calculated the proportions for each response type (see Table 1 for details).

**TABLE 1:** Average response proportions for different response types in picture naming and retrieval failure of semantics and phonology.

**Response**	**Young adults**		**Older adults**	
	***M* (%)**	***SE* (%)**	***M* (%)**	***SE* (%)**
pTOT	7.51	1.40	20.56	1.40
GOT	34.21	1.80	17.07	1.80
DK	19.32	2.10	34.85	2.10
Step 1 retrieval failure (Semantic retrieval deficit)	58.28	2.80	59.05	2.80
Step 2 retrieval failures (Phonological retrieval deficit)	16.36	2.40	52.91	2.40

*pTOT, positive tip-of-the-tongue; GOT, correct naming of the target picture; DK, don’t know.*

In order to exclude the potential influence of general inhibitory deficit, which presented the age-related difference in Stroop task (*F* (1, 70) = 11.024, *p* = 0.001, η*_*p*_*^2^ = 0.136), we conducted an analysis of covariance (ANCOVA) on three response types (pTOT, GOT, DK) with age as a between-participant variable and the net Stroop effect (incongruent RT – congruent RT) as a covariant. The results for pTOTs revealed a significant main effect for age after controlling the age-related inhibitory deficit, *F*1 (1, 69) = 46.881, *p* < 0.001, η_*p*_^2^ = 0.405, *F*2 (1, 216) = 7.019, *p* = 0.009, η*_*p*_*^2^ = 0.031, showing more TOTs for older than young adults. There were more GOTs for young adults, and age effect was significant, *F*1 (1, 69) = 34.345, *p* < 0.001, η*_*p*_*^2^ = 0.332, *F*2 (1, 216) = 22.672, *p* < 0.001, η*_*p*_*^2^ = 0.095. The analysis of DKs also reported a main effect for age, *F*1 (1, 69) = 20.374, *p* < 0.001, η_*p*_^2^ = 0.228, *F*2 (1, 216) = 24.769, *p* < 0.001, η*_*p*_*^2^ = 0.103, showing more DKs for older than young adults. Negative TOT and FOK were of no interest to the current study (Pureza et al., 2013).

According to the two-step approach, Step 1 and Step 2 retrieval failures reflect that speakers do not fully retrieve semantic and phonological information of the target name, respectively. ANCOVAs were performed on the proportion of responses that reflect semantic (step 1) and phonological (step 2) retrieval failures with net Stoop effect as a covariate. For step 1, the main effect of age was not significant, *F*1 (1, 69) < 1, *p* > 0.05, η*_*p*_*^2^ < 0.001, *F*2 (1, 216) = 14.935, *p* < 0.001, η*_*p*_*^2^ = 0.065. Whereas for step 2, the main effect of age was significant, *F*1 (1, 69) = 105.868, *p* < 0.001, η*_*p*_*^2^ = 0.605, *F*2 (1, 216) = 33.705, *p* < 0.001, η*_*p*_*^2^ = 0.135, showing a higher proportion of phonological retrieval deficit for older than young adults.

### Discussion

Using a two-step approach in both young and older groups, we obtained three important findings. First, in accordance with previous studies (Burke et al., 1991; Evrard, 2002; Cross and Burke, 2004; Hanley, 2011), aging influenced TOT occurrence by showing more TOTs for proper names in older adults. Second, compared with young adults, older adults exhibited greater deficit in phonological retrieval but similar magnitude in semantic retrieval during lexical access, which is strongly consistent with the TDH’s assumption. Most importantly, these findings remained unchanged even after canceling out age-related difference in inhibitory efficiency, which highlight a unique role of information-specific deficit in aging difference in spoken word production. The finding that semantic retrieval did not vary with age is inconsistent with Juncos-Rabadán et al.’s (2010). One possibility for this different pattern arises from the different measures of semantic retrieval. Juncos-Rabadán et al. (2010) provided a novel measure calculated by (GOTs + pTOTs + negative TOTs)/N, which covers the semantic retrieval that does not correspond to semantic representations of targets as in negative TOTs and therefore might overestimate the semantic retrieval during lexical access. Altogether, our study provides convincing evidence that age-related increase in TOTs for proper names was mainly due to phonological retrieval failures rather than semantic retrieval failures (Burke et al., 1991; Rastle and Burke, 1996; Evrard, 2002; Gollan and Brown, 2006; Juncos-Rabadán et al., 2010).

## Experiment 2

The main goal of this experiment was to test whether semantic and phonological information retrievals can be facilitated by semantic and phonological priming, and further compare the age-related difference in the priming effect during names access. In experiment 2 with a priming paradigm, participants were presented semantically related or phonologically related names before target pictures, which formed bottom-up semantic or phonological priming conditions. The relationship between prime names and target names was manipulated in term of semantic and phonological relatedness. For semantic relatedness, the prime names belonged to the same occupation as the target names or did not belong to the same occupation. For phonological relatedness, the prime names shared the first names, or the first syllables with the target names, or unrelated to the target names.

### Methods

#### Participants

Participants were 82 individuals who have participated in experiment 1.

#### Materials and Design

One hundred and twenty photographs of the faces of famous persons from different professions (e.g., actor, singer, TV host, sportsman, politician, scholar, and businessman) were selected as target pictures. The pictures here were entirely different from those used in experiment 1. Each target picture was paired with six different types of prime words, including 2 types of semantically relatedness condition and 3 types of phonologically relatedness condition, two factors manipulated orthogonally. A semantically plus first-name related prime word shared the same occupation and the first name with target name (e.g., 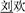,/liu2huan1/, being a singer as a target; 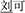,/liu2ke2/, being a singer as a prime. The first names are underlined). A semantically plus first-syllable related prime word shared the same occupation and the same syllable of the first name with target name (e.g., 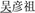,/wu2yan4zu3/, being an actor as target; 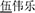,/wu3wei2le4/, being an actor as a prime.). A semantically related but phonologically unrelated prime word shared the same occupation but had no phonological overlap with the target name (e.g., 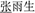,/zhang1yan4zu3/, being a singer as target, 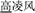,/gao1ling2feng1/, being a singer as a prime.). A semantically unrelated but the first-name related prime word did not share the same occupation but shared the first name with target name (e.g., 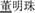,/dong3ming2zhu1/, being a businessman as target, 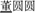,/dong3yuan2yuan2/, being an actress as a prime). A semantically unrelated but the first-syllable related prime word name did not share the same occupation but shared the first syllable with target name (e.g., 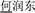,/he3run4dong1/, being an actor as target, 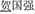,/he4guo2qiang2/, being a politician as a prime). A semantically unrelated plus phonologically unrelated prime word did not share the same occupation and had no phonological relation with target name (e.g., 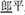,/lang2ping2/, being a sportsman as target, 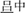,/lv3zhong1/, being an actress as a prime) (see Table 2). The materials used were presented in Supplementary Appendix B.

**TABLE 2:** Examples of target and prime pairs used in experiment 2, and the means familiarity rates and the number of syllables of the pairs.

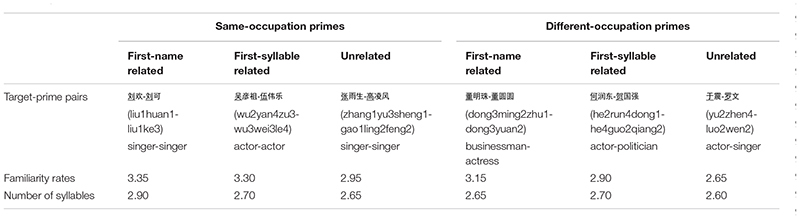

The experiment design included age (young vs. old), semantic relatedness (same occupation vs. different occupation) and phonological relatedness (the first-name related, the first-syllable related, and unrelated). For each age group, 120 trials were evenly divided into six experimental conditions. The order of target pictures was pseudo-randomize to prevent target pictures with the same manipulation from repeating across three trials.

To investigate the possible difference in target pictures among the experimental conditions, twelve young and twelve older adults who did not take part in the formal experiment named and rated their familiarity of picture stimuli on a 5-point Likert scale (1 = *very low*; 5 = *very high*). The familiarity for young adults (*M* = 2.96 ± 0.31) and older adults (*M* = 3.12 ± 0.27) was matched, *t* (22) = –1.32, *p* = 0.20. The familiarity of target pictures was matched between semantically related and unrelated conditions, *t* (118) = 1.689, *p* = 0.093, and among three phonologically related conditions, *F* (2, 114) = 2.246, *p* = 0.111. The interaction between semantic relatedness and phonological relatedness was not significant as well, *F* (2, 114) < 1, *p* = 0.899. Furthermore, we analyzed the number of syllables for target names in different conditions, and revealed non-significant effects between semantically related and unrelated conditions, *t* (118) = 1.192, *p* = 0.236, and among different phonological related conditions, *F* (2, 114) = 1.064, *p* = 0.348, and their interaction, *F* (2, 114) < 1, *p* = 0.44.

#### Procedure

The procedure was similar to experiment 1 except that participants performed a pronunciation difficulty rating task containing six types of prime names before the presentation of target pictures. The prime name was presented visually on the white computer screen for 3000 ms, and then participants had to rate how easily the prime could be pronounced using a 3-point scale (1 = *easy*, 3 = *hard*) (Rastle and Burke, 1996; James and Burke, 2000; Abrams et al., 2007). After rating the prime name, a paired picture was presented immediately and participants were required to name the picture or to make a button response. The procedure was identical to experiment 1, with a consistent correspondence between response type and button response. The priming procedure could keep participants unaware of the relationship between primes and target words (see also White and Abrams, 2002 for a similar manipulation).

### Results

Four young adults and three older adults were excluded because their percentage of pTOTs was less than three standard deviations from cell means. Data from incorrect responses (1.04% and 0.88% for young and older participants, respectively) were removed from all analyses. For each participant, we calculated the proportions for each response type (see Table 3 for details). Response performance was recorded for each trial, and each response proportion was analyzed using a 2 × 2 × 3 repeated measures ANCOVAs similar to experiment 1. Especially, we used semantic relatedness and phonological relatedness as within-subjects variables, and age as a between-subjects variable (*F*1), and semantic relatedness and phonological relatedness as between-items variables and age as within-items variable (*F*2). To investigate whether the feedback links between phonological and semantic representational levels when TOTs occur are subjected to age-related impairments as the forward links do, we analyzed the proportions of pTOTs, semantic retrieval deficit, and phonological retrieval deficit in further analyses.

**TABLE 3:** Average response proportions (*M* ± *SE*) for target pictures by age, semantic relatedness, and phonological relatedness.

		**Same-occupation**			**Different-occupation**		
			
		**First-name related**	**First-syllable related**	**Unrelated**	**First-name related**	**First-syllable related**	** Unrelated**
pTOT	Young	0.038 ± 0.008	0.068 ± 0.016	0.066 ± 0.015	0.077 ± 0.015	0.073 ± 0.015	0.071 ± 0.015
	Older	0.077 ± 0.008	0.159 ± 0.016	0.177 ± 0.015	0.191 ± 0.015	0.194 ± 0.015	0.164 ± 0.015
Semantic retrieval deficit	Young	0.464 ± 0.032	0.464 ± 0.032	0.728 ± 0.036	0.425 ± 0.041	0.614 ± 0.034	0.642 ± 0.038
	Older	0.646 ± 0.033	0.658 ± 0.034	0.689 ± 0.030	0.468 ± 0.030	0.600 ± 0.026	0.623 ± 0.026
Phonological retrieval deficit	Young	0.096 ± 0.022	0.100 ± 0.010	0.250 ± 0.040	0.130 ± 0.024	0.159 ± 0.026	0.179 ± 0.032
	Older	0.339 ± 0.034	0.494 ± 0.040	0.690 ± 0.040	0.430 ± 0.029	0.492 ± 0.034	0.479 ± 0.041

*pTOT, positive tip-of-the-tongue.*

#### Positive TOT Proportion

We conducted a three-way ANCOVA on pTOTs proportion, with participants (*F*1) or items (*F*2) as random factors, semantic relatedness and phonological relatedness as within-subjects variables (or between-items), and age as a between-subjects variable (or within-items). Table 4 presents the results of pTOTs proportion after excluding the influence of inhibition ability via using Stroop Color-Word task measures as a covariate in ANCOVA analysis. We observed significant main effects of age, semantic relatedness and phonological relatedness, showing that less TOTs were induced for young than older adults, for same-occupation than different-occupation primes, and for the first-name or the first-syllable related primes than unrelated primes.

**TABLE 4:** ANCOVA of the proportions of the pTOT, semantic retrieval deficit, and phonological retrieval deficit by age, semantic relatedness (SR), and phonological relatedness (PR).

**Source**	***F***	**pTOT**				**Semantic retrieval deficit**				**Phonological retrieval deficit**			
				
		***MSE***	***F*1*/F*2**	***p***	**η*_*p*_^2^***	***MSE***	***F*1*/F*2**	***p***	**η*_*p*_^2^***	***MSE***	***F*1*/F*2**	***p***	**η*_*p*_^2^***
Age	*F*1(1,70)	3.744	37.099	< 0.001	0.350	0.250	0.852	0.359	0.012	14.674	111.242	< 0.001	0.617
	*F*2(1,114)	0.887	71.392	0.001	0.238	0.230	2.550	0.081	0.020	8.533	352.336	< 0.001	0.607
SR	*F*1(1,69)	0.800	23.14	< 0.001	0.251	0.216	12.915	0.001	0.158	< 0.001	0.306	0.582	0.004
	*F*2(1,114)	0.060	4.794	0.030	0.020	0.299	4.363	0.038	0.019	0.003	0.137	0.712	0.001
PR	*F*1(2,138)	0.029	5.299	0.007	0.071	0.342	61.319	< 0.001	0.471	0.222	10.300	< 0.001	0.130
	*F*2(2,114)	0.960	6.348	0.002	0.053	0.361	5.259	0.006	0.044	0.538	22.213	< 0.001	0.163
Age × SR	*F*1(1,69)	0.267	5.736	0.019	0.077	0.461	24.139	< 0.001	0.259	0.012	1.831	0.180	0.026
	*F*2(1,114)	0.018	1.429	0.233	0.006	0.402	5.869	0.016	0.025	0.026	1.085	0.299	0.005
Age × PR	*F*1(2,138)	0.014	2.540	0.085	0.036	0.311	25.818	< 0.001	0.272	0.099	3.044	0.050	0.042
	*F*2(2,114)	0.366	0.366	0.694	0.003	0.297	4.336	0.014	0.037	0.100	4.123	0.017	0.035
SR × PR	*F*1(2,138)	0.196	10.097	< 0.001	0.128	0.178	18.402	< 0.001	0.211	0.103	6.685	0.002	0.088
	*F*2(2,114)	0.042	3.386	0.036	0.029	0.219	3.188	0.043	0.027	0.066	2.725	0.068	0.023
Age × SR × PR	*F*1(2,138)	0.151	4.869	0.009	0.066	0.166	14.114	< 0.001	0.170	0.067	3.236	0.042	0.045
	*F*2(2,114)	0.009	0.801	0.451	0.014	0.060	1.223	0.298	0.021	0.018	2.067	0.131	0.035

**FIGURE 3:**
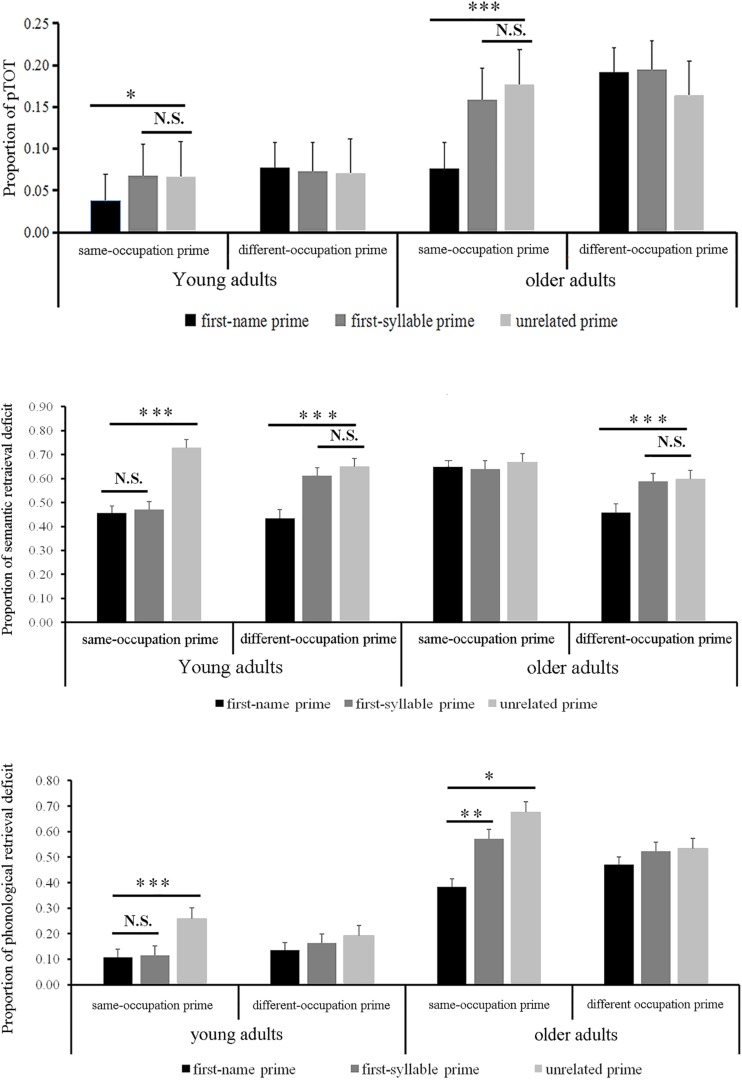
Proportions of pTOT (upper panel), semantic retrieval deficit (middle panel), and phonological retrieval deficit (lower panel) as a function of semantic relatedness and phonological relatedness in young and older adults in Experiment 2. Vertical bars correspond to standard errors. Asterisks indicate significance of the difference (^∗^*p* < 0.05; ^∗∗^*p* < 0.01; ^∗∗∗^*p* < 0.001; and N.S., *p* > 0.05).

The most important finding is a three-way interaction between age, semantic relatedness and phonological relatedness as illustrated in Figure 3 (upper panel). Further analyses showed that the two-way interaction between semantic relatedness and phonological relatedness was modulated by age. More precisely, ANCOVA for young adults showed a marginal interaction effect between the two variables, *F*1 (2, 68) = 3.076, *p* = 0.056, η*_*p*_*^2^ = 0.083, *F*2 (2, 114) = 2.232, *p* = 0.112, η*_*p*_*^2^ = 0.038, where for same-occupation primes, fewer TOTs occurred when the primes were also the first-name related than the first-syllable or unrelated. But ANCOVA for older adults showed a strong interaction effect between the two variables, *F*1 (2, 68) = 6.693, *p* = 0.002, η*_*p*_*^2^ = 0.164, *F*2 (2, 114) = 2.009, *p* = 0.139, η*_*p*_*^2^ = 0.034, where for same-occupation primes, much fewer TOTs occurred when the primes were also the first-name related than the first-syllable related or unrelated.

#### Semantic Retrieval Deficit

We conducted a similar analysis on semantic retrieval deficit (see Table 4 middle column). The significant main effect of semantic relatedness suggested a semantic retrieval advantage for different-occupation primes. The significant main effect of phonological relatedness also indicated a facilitation effect in the first-name related or the first-syllable related primes. The two-way interaction between age and semantic relatedness, the one between age and phonological relatedness, and the one between semantic relatedness and phonological relatedness were significant.

The three-way interaction between age, semantic relatedness and phonological relatedness was significant (see Figure 2 middle panel). We analyzed this interaction using separate ANCOVAs for young and older adults. For young adults, a significant interaction between semantic relatedness and phonological relatedness was observed, *F*1 (2, 68) = 18.639, *p* < 0.001, η*_*p*_*^2^ = 0.354, *F*2 (2, 114) = 2.232, *p* = 0.102, η*_*p*_*^2^ = 0.039. Further analysis showed that semantic retrieval deficit decreased significantly when the prime was also the first-name related or the first-syllable related than unrelated in same-occupation primes condition, and when the prime was also the first-name related than the first-syllable related and unrelated primes in different-occupation primes. For older adults, the interaction between semantic relatedness and phonological relatedness was significant, *F*1 (2, 68) = 9.143, *p* = 0.001, η*_*p*_*^2^ = 0.212, *F*2 (2, 114) = 2.274, *p* = 0.108, η*_*p*_*^2^ = 0.038, further analysis showed that semantic retrieval deficit decreased significantly when comparing the first-name related with the first-syllable related or unrelated in different-occupation primes condition. None of them was significant in same-occupation primes.

#### Phonological Retrieval Deficit

We conducted a three-way ANCOVA on phonological retrieval deficit (see Table 4 right column). The effect of age was significant, reflecting a phonological retrieval advantage in young adults. The effect of phonological relatedness was significant, reflecting participants produced a larger priming effect in the first-name related or in the first-syllable related primes condition when compared with unrelated condition. The two-way interaction between age and phonological relatedness and the one between semantic relatedness and phonological relatedness were significant.

Our principal interest here is the borderline significant three-way interaction between age, semantic relatedness and phonological relatedness (see Figure 2 lower panel). To decompose this complex interaction, we conducted separate ANCOVAs for young and older adults. For young adults, the interaction between semantic relatedness and phonological relatedness was significant, *F*1 (2, 68) = 3.414, *p* = 0.039, η*_*p*_*^2^ = 0.091, *F*2 (2, 114) = 4.701, *p* = 0.011, η*_*p*_*^2^ = 0.076, where phonological retrieval deficit, for same-occupation primes, was smaller when the prime was the first-name related or the first-syllable related than the unrelated condition, but the former two phonologically related conditions did not differ significantly (*p* = 0.841). For older adults, the significant interaction was observed as well, *F*1 (2, 68) = 3.563, *p* = 0.034, η*_*p*_*^2^ = 0.095, *F*2 (2, 114) = 1.953, *p* = 0.147, η*_*p*_*^2^ = 0.033, where phonological retrieval deficit, for same-occupation primes, was smaller when the prime was the first-name related or the first-syllable related than unrelated, and the former two phonologically related conditions differed significantly (*p* = 0.004). Both groups did not present any significant differences among three phonological relatedness conditions in different-occupation primes.

### Discussion

Results showed that older adults produced more TOTs and greater phonological retrieval deficit for proper names than young adults, whereas both groups had similar semantic retrieval deficit, which replicated experiment 1’s finding and were consistent with previous studies (Cross and Burke, 2004; Thornton and Light, 2006; Burke and Shafto, 2008; Hanley, 2011). Critically, experiment 2’s results demonstrate important age differences in the bottom-up semantic and phonological priming effects on TOTs.

For the proportion of TOTs, the most important finding is a three-way interaction between age, semantic relatedness and phonological relatedness, presenting a strong interaction between semantic relatedness and phonological relatedness in older adults while a marginal one in young adults. Further analysis showed that the facilitatory effect of phonological priming was more prominent for older adults than young adults, indicating an age-related change in the effect of phonological priming on TOTs occurrence. Furthermore, this effect was significant in same-occupation primes but not in different-occupation primes in both groups, reflecting a symmetric semantic priming effect in TOTs occurrence.

For semantic retrieval deficit, a significant three-way interaction among three variables indicated that the interaction between semantic and phonological relatedness was modulated by age. To be specific, older adults presented comparable semantic retrieval failures relative to young adults. Furthermore, phonologically related primes could help speakers reduce TOTs at the lexico-semantic level. That is, phonologically related primes improve targets’ semantic activation via a bottom-up pathway from phonology to semantics. Specifically, older adults require a larger phonological overlap relation between primes and targets (i.e., the first-name related) while young adults require a less phonological overlap (i.e., the first-syllable related), in reducing TOTs occurrence, reflecting a weaker connection between semantics and phonology in older adults than young adults. Older adults presented phonological effect in different-occupation condition but not in same-occupation condition, while young adults presented similar phonological effects in both occupation primes, indicating a larger semantic interference effect in older adults in comparison with young adults. These findings indicated that the bottom-up semantic and phonological priming influence targets activation at the semantic level in both groups.

For phonological retrieval deficit, a significant three-way interaction among three variables indicated that the interaction between semantic and phonological relatedness was modulated by age. Furthermore, both young and older adults produced larger phonological facilitation effect in same-occupation condition, but not in different-occupation condition, indicating similar bottom-up phonological priming effect in reducing TOTs occurrence. In addition, phonological facilitation was disproportional for different priming conditions in older adults compared with young adults, indicating that older adults are more sensitive to phonological priming in reducing TOTs than young adults. These findings were in line with the TDH’s hypothesis that the age-related difference is due to the weakened connection between semantics and phonology in older adults than young adults.

## General Discussion

Experiments 1 and 2 yielded important results that support asymmetric aging effects on TOTs occurrence, semantic retrieval deficit and phonological retrieval deficit during lexical access. Note that we excluded the influence of general inhibition ability in the data analysis. First, older adults reported more TOTs in person naming and more phonological retrieval deficit in TOTs occurrence than young adults, while both groups shown comparable semantic retrieval deficit. Second, for semantic retrieval deficit, we observed a larger interference effect for older than young adults in bottom-up semantic priming and a smaller phonological facilitation effect for older than young speakers in bottom-up phonological priming. Third, for the phonological retrieval deficit, there was an age difference in bottom-up phonological priming, but not in bottom-up semantic priming. Overall, our findings are best understood in the theoretical framework of the TDH, in which aging enriches semantic representations but causes deficit in the specific connection between semantics and phonology in speaking.

### Age Difference in TOTs in Bottom-Up Semantic and Phonological Priming

By comparing experiments 1 and 2, we found that TOTs occurrence decreased in bottom-up semantic and phonological priming conditions, suggesting that semantically or phonologically related primes facilitate lexical retrieval of target names, which is consistent with previous findings in proper names production (Rastle and Burke, 1996; Burke et al., 2004; Oberle and James, 2013) and common nouns production (James and Burke, 2000; Burke et al., 2004; Kavanaugh, 2015). According to the TDH, the decreased phonological activation of target names results in more TOTs in older adults. Thus, it is plausible that the phonologically related primes improve phonological activation of target names and reduce TOTs occurrence. By contrast, the finding that semantically related primes facilitated targets retrieval and then reduced TOTs occurrence was in line with previous studies (Rastle and Burke, 1996; Cross and Burke, 2004; Farrell, 2012; Oberle and James, 2013). Oberle and James (2013) found that prior presentation of a semantically related name which shared the occupation with the target could reduce TOTs occurrence and increase correct retrieval of a pictured person. However, semantic priming effects in TOTs were inconsistent in the literature. Farrell (2012) found that semantic primes did not influence TOTs of common names. One possibility for the difference is the materials used in studies. Unlike common names, proper names possess a relatively meaningless, arbitrary and non-descriptive semantic system (Cohen, 1990; Evrard, 2002; Burke et al., 2004), which may result in semantic retrieval deficit as well. Semantically related primes could strengthen connections among semantic representations of the word to be retrieved in the subsequent picture and then facilitate its semantic retrieval. In short, the facilitatory effects of semantically or phonologically related primes indicate that proper names are particularly susceptible to retrieval failures not only for phonological information, but also for semantic information.

We are also interested in age differences in the benefits from both semantic and phonological priming, which allow us to examine age-related declines in the transmission of activation between semantic and phonological representations. We observed that the facilitatory effect of phonological priming was more prominent for older adults than young adults. This is because proper names are particularly vulnerable to transmission deficit and the deficit is more serious in older adults. The finding of age-related differences in the influence of phonologically related primes on TOT occurrences is inconsistent with studies which found similar priming effects for both age groups (Rastle and Burke, 1996; James and Burke, 2000; Cross and Burke, 2004; Farrell, 2012). One possibility for the absence of age difference in those studies was that participants were unaware of the phonological priming manipulation involved (Burke et al., 2004). Evidence reveals that older adults would show a phonological priming effect on TOTs occurrence only after excluding the influence from conscious awareness (Burke et al., 2004). In the present study, young adults reported that they noticed the phonological relationship between prime words and target names, whereas older adults seemed naïve to this manipulation because they were less able to utilize conscious recollection strategies (Light and La Voie, 1993; Titov and Knight, 1997). In addition, a recent study using picture-word interference paradigm reported that older but not young adults benefited from phonological distractor to a greater extent for the production of low-frequency targets than high-frequency targets (Rizio et al., 2017). The authors attributed this finding to age-related transmission deficit that differentially affect low-frequency versus high-frequency words. Considering that proper names are characterized by low frequency or long periods of non-use, the finding of better performance in TOT reductions for older adults suggests that proper names are susceptible to transmission deficit advancing with age. This age-related asymmetry in phonological priming effect on TOTs occurrence supports the view that age is a unique factor contributing to retrieval failures of proper names.

Interestingly, we observed that older adults also benefited more from semantic priming to decrease TOTs occurrence than young adults, which contradicted with the TDH’s assumption. However, Rastle and Burke (1996, experiment 2) found that prior semantic processing (i.e., pleasantness rating) reduced TOTs occurrence much more for older than young adults. Processing a semantically related word may produce facilitation or interference in a picture naming task, depending on the nature of the semantic relation and the onset interval between primes and targets. Studies have demonstrated that facilitation effect occurs at the conceptual level while interference effect occurs at the lexical level (Finkbeiner and Caramazza, 2006). Furthermore, transmission of priming in the semantic system is realized by multiple connections that link lexical nodes for semantically related concepts (MacKay, 1987). We thus suggested that semantic facilitation effects here arose at the conceptual level. There is a richer semantic network in older adults than young adults, which results in a larger semantic priming effect in reducing TOTs occurrence. Our findings were also consistent with age-related semantic priming effects in word recognition (Cerella and Fozard, 1984; Bowles and Poon, 1988).

### Age Difference in Semantic Retrieval Deficit

Both experiments provided consistent evidences that more TOTs in older adults were not caused by age-related semantic retrieval deficit (see also Gollan and Brown, 2006; Juncos-Rabadán et al., 2010). In addition, semantically related primes exerted an adverse effect on semantic retrieval when TOTs occurred, but this effect was confined to older adults. Studies have reported that semantically related primes decreased TOT resolution rates and even blocked retrieval in older speakers (Abrams and Rodriguez, 2005; Abrams et al., 2007; White et al., 2013). Semantically related primes interfere with targets retrieval if they compete for lexical selection, as evidenced by semantic interference effect (Damian et al., 2001; Zhang et al., 2018). Older adults showed greater semantic interference and priming effects in comparison with young adults (Laver and Burke, 1993; Taylor and Burke, 2002; Rizio et al., 2017). For the above reason, we speculate that older speakers decline in their ability to ignore irrelevant information, and thus semantically related primes are more likely to block semantic retrieval during lexical access.

An interesting finding was that phonologically related prime words facilitated semantic retrieval, and critically young adults benefited more from phonological priming on semantic retrieval than older adults. The TDH postulates that top-down connection from semantics to phonology is more vulnerable to age-related transmission deficit than bottom-up connection. However, recent findings revealed that aging could weaken all potential connections, resulting in deficits in both top-down and bottom-up activation transmission for written language comprehension (Robert and Mathey, 2007; Shafto, 2010; Robert and Duarte, 2016). According to the interactive activation models of spoken word production (Dell, 1986), a prime activates its phonological representation and then the activation could feedback from phonology to lemmas via shared phonological information. In the current study, phonologically related primes strengthen the connection from phonology to semantics which would improve the semantic activation of target names and enhance targets semantic retrievals. Our findings firstly indicated an age difference from phonology-to-semantics in spoken word production, and thus have strong implications for the TDH.

For the semantic retrieval deficit, older adults presented a phonological priming effect in same-occupation primes but not in different-occupation primes, in comparison with young adults, suggesting that the magnitude of phonological priming effect for older adults is constrained by semantic contexts. There is evidence showing that the phonological priming of lexical retrieval after TOTs occurrence is constrained by semantic category (White et al., 2013) or grammatical class between primes and target names, and older adults are more sensitive to these modulations of semantic contexts than young ones (Abrams and Rodriguez, 2005; Abrams et al., 2007). We also observed an age-related difference in the phonological priming effect on lexical retrieval during TOTs occurrence, depending on the semantic constrains between primes and target names. These age-related differences in pattern of phonological priming effect dependent on the semantic relatedness contribute to our understanding of the interactivity between the semantic and phonological representations, and how age-related change affects this interactivity.

### Age Difference in Phonological Retrieval Deficit

Both experiments converge to the finding that older adults exhibited significantly more phonological retrieval failures than young adults (Levelt et al., 1999; Gollan and Brown, 2006). Additionally, we observed a facilitatory effect on phonological retrieval only from bottom-up phonological priming but not semantic priming. Mounting evidence confirmed the effectiveness of phonological priming in improving the target retrieval both before and after TOT occurrence (James and Burke, 2000; White and Abrams, 2002; Burke et al., 2004; Pureza et al., 2013; White et al., 2013). To account for this phenomenon, researchers postulate that phonological priming strengthens the connections between semantic and phonological nodes, and eventually facilitates phonological retrieval. White et al. (2013) compared the effect of two types of phonologically related primes on TOT resolution and found greater phonological priming for same-name primes than same-syllable primes. Here, we extended those findings and demonstrated that age has an important role in the magnitude of facilitation effect on phonological retrieval. Older adults produced significant more priming effect in the first-name related condition compared with the first-syllable related condition, whereas young adults produced comparable priming effects in the two related conditions. We think that the phonological priming of the first-syllable related primes on phonological retrieval was not strong enough as that of same-name primes for older adults given that same-syllable priming could not completely offset age-related declines in the connection deficit. These findings are inconsistent with the prediction based on the incomplete activation hypothesis (Brown and McNeill, 1966), since phonological retrieval is sensitive to phonological priming (Mirman and Britt, 2014), especially for older adults. Taken together, we provide a direct evidence for age-related difference in the phonological priming effect for phonological retrievals, which indicates that aging weakens the connections between semantics and phonology.

Studies have revealed that the phonological priming was sensitive to age, and the critical age of divergence differed across various samples (White and Abrams, 2002; Abrams et al., 2007). For example, White and Abrams (2002) showed that young and young-old adults (60–72 years old) benefited similarly from the first-syllable related primes in TOTs resolution, but old-old adults (73–83 years old) showed no increase in TOTs resolution following prime words. However, using a similar priming task, Abrams et al. (2007) observed that young-old adults (61–73 years old) benefited less from the first-syllable related primes in TOTs resolution than young adults, while old-old adults (75–89 years old) did not produce priming effect in the first-syllable related primes but instead showed an inhibitory effect in TOTs resolution after the prime words presentation. In the present study, we extended this finding to TOTs occurrence by showing that older adults aged from 60–72 years exhibited the first-syllable related priming to a lesser degree than younger adults, which is consistent with Abrams et al.’s (2007) finding (but see White and Abrams, 2002).

Studies demonstrated that the relation between semantics and phonology is discrete in Chinese but interactive in alphabetic languages such as English (Starreveld and La Heij, 1995; Damian and Martin, 1999; Zhu et al., 2015, 2016; Zhang et al., 2018), which might lead to different patterns of aging effect across language systems. In the present study, however, we observed a significant interaction between factors of semantic and phonological relatedness when target names were not retrieved successfully, reflecting an interactive pattern of semantics and phonology in reducing TOTs occurrence. However, we suggest that this finding is not contradicted with a discrete pattern in a typical successful picture naming. When TOTs occur, speakers have experienced a naming process and have retrieved partial semantic and phonological information, so it is plausible to present an interactive pattern here.

Alternatively, previous studies have showed that the activation flow between the conceptual representations, lexical (i.e., semantic) representations and phonological representations is proved to be modulated by factors such as allocation and availability of processing resources (Mädebach et al., 2011), frequency trajectory of target words (Bonin et al., 2012). In the theoretical framework of the discrete hypothesis, it has been generally accepted that there is some interactive (limited-cascading) within the lexical system in word production (Kuipers and Heij, 2009; Dumay and Damian, 2011). In other words, information flow in spoken word production is task-dependent or goal-related (Roelofs, 1997). Therefore, we speculate that the significant interaction between semantic and phonological relatedness may indicate that the activation flow between word’s semantic and phonological representations is bidirectional during proper name access, which is consistent with the argument of the interactive activation models.

### Aging Difference in the Connections Between the PINs and Phonological Nodes

As outlined in the introduction, production of proper names might involve parallel access to semantic and phonological nodes from PINs via bi-directional links between them (Valentine et al., 1996). The semantic and phonological priming effects on reducing TOTs occurrence are consistent with the predictions derived from the functional model of face naming (Valentine et al., 1996). As a type of speech error, TOT is regarded as a retrieval failure for phonological rather than semantic information (Burke et al., 1991). Prior presentation of semantic primes may increase the activation of PINs which in turn strengthen the top-down connection from PINs to phonological nodes, ultimately facilitating the phonological retrieval of target name. Similarly, phonological primes may reactivate PINs via bottom-up connections, and then active PINs which spread activation to phonological nodes. Following similar logics, the semantic and phonological priming effects on semantic and phonological retrievals are plausible in the face-naming models.

Importantly, the present results showed that older adults compared with young ones benefited less from phonological priming effect on reducing TOTs occurrence and facilitating phonological retrieval, suggesting that the connections between PINs and phonological nodes might be weaker in older adults than in young adults. Results of age-related difference in phonological priming on semantic retrieval also support this notion. Valentine et al. (1995) reported that making occupation decision or reading aloud the surname of a visually presented full name of a celebrity facilitated subsequent face naming on seeing the celebrity, and they interpreted that priming from prior name recognition increased the weight of the link between the PINs and lexical output code (i.e., phonological nodes) (see also Johnston and Barry, 2006). The present study confirms and extends the findings in these studies by showing that the connections between the PINs and phonological nodes may be sensitive to normal aging, which is similar to the connections between semantic and phonological nodes as discussed above. However, we cannot distinguish the PINs from semantically or phonologically related primes in the present study, and it is necessary to separate these variables to investigate the relations between the PINs and semantic or phonological nodes in future studies.

## Conclusion

In sum, when speakers produce proper names, age-related increase in TOTs occurrence are due to the decline in phonological retrieval rather than semantic retrieval in older adults. For semantic retrieval deficit, older adults exhibited smaller phonological facilitation effect and a larger semantic interference effect than young adults. For phonological retrieval deficit, older adults presented a smaller phonological facilitation effect than young adults, whereas both groups presented comparable semantic priming effect. Overall, our findings provide evidence for the transmission deficit hypothesis from the bottom-up phonological and semantic priming. The influence of general inhibition ability for different age groups was excluded in the present study, thus we suggest that the age difference in TOTs occurrence is information-specific to a certain degree.

## Data Availability Statement

The datasets generated for this study are available on request to the corresponding author.

## Ethics Statement

The current study was reviewed and approved by the Ethics Committee of the Department of Psychology, Renmin University of China. Written consents were obtained from participants before the administration of experiments.

## Author Contributions

MO and QZ conceived and designed the experiment. MO performed the experiment and analyzed the data. MO, XC, and QZ interpreted the results and wrote the manuscript. All authors read and approved the final manuscript.

## Conflict of Interest

The authors declare that the research was conducted in the absence of any commercial or financial relationships that could be construed as a potential conflict of interest.
