# Supplementary material for: Aging Effects on Phonological and Semantic Priming in the Tip-of-the-Tongue: Evidence From a Two-Step Approach
Source: Front Psychol. 2020 Feb 27;11:338. doi: 10.3389/fpsyg.2020.00338 (PMC7056892; doi:10.3389/fpsyg.2020.00338)
Supplement: Supplementary file 1 [file Data_Sheet_1.doc]

| Appendix A: Stimuli used in experiment 1. | | | | | |
| --- | --- | --- | --- | --- | --- |
| Target picture names | Pinyin | Occupations | Target picture names | Pinyin | Occupations |
| 李嘉诚 | li2jia1cheng2 | businessman | 林俊杰 | lin2jun4jie2 | singer |
| 马云 | ma3yun2 | businessman | 谢霆锋 | xie4ting2feng1 | singer |
| 史玉柱 | shi3yu4zhu4 | businessman | 张国荣 | zhang1guo2rong2 | singer |
| 陈浩民 | chen2hao4min2 | actor | 郑少秋 | zheng4shao4qiu1 | actor |
| 樊少皇 | fan2shao4huang2 | actor | 陈奕迅 | chen2yi4xun4 | singer |
| 李若彤 | li2ruo4tong2 | actress | 张智霖 | zhang1zhi4lin2 | actor |
| 梁烈唯 | liang2lie4wei2 | actor | 齐秦 | qi2qin2 | singer |
| 梁靖琪 | liang2jing4qi2 | actress | 濮存昕 | pu2cun2xin1 | actor |
| 萧亚轩 | xiao1ya4xuan1 | singer | 庾澄庆 | yu2cheng2qing4 | singer |
| 夏雨 | xia4yu3 | actor | 乔仁梁 | qiao2ren2liang2 | singer |
| 惠英红 | hui4ying1hong2 | singer | 古巨基 | gu3ju4ji1 | singer |
| 邓超 | deng4chao1 | actor | 林志炫 | lin2zhi4xuan4 | singer |
| 郭晓冬 | guo1xiao3dong1 | actor | 光良 | guang1liang2 | singer |
| 谢天华 | xie4tian1hua2 | actor | 莫文蔚 | mo4wen2wei4 | singer |
| 徐少强 | xu2shao4qiang2 | actor | 范玮琪 | fan4wei3qi2 | singer |
| 洪金宝 | hong2jin1bao3 | actor | 梁静茹 | liang2jing4ru2 | singer |
| 元彪 | yuan2biao1 | actor | 黄渤 | huang2bo2 | actor |
| 陈志朋 | chen2zhi4peng2 | singer | 刘蓓 | liu2bei4 | actress |
| 曾志伟 | zeng1zhi4wei3 | actor | 吕丽萍 | lv3li4ping2 | actress |
| 吴樾 | wu2yue4 | actor | 张曼玉 | zhang1man4yu4 | actress |
| 邹兆龙 | zou1zhao4long2 | actor | 张铁林 | zhang1tie2lin2 | actor |
| 赵文卓 | zhao4wen2zhuo2 | actor | 丁宁 | ding1ning2 | sportsman |
| 刘涛 | liu2tao1 | singer | 张继科 | zhang1ji4ke1 | sportsman |
| 赵丽颖 | zhao4li4ying3 | singer | 马龙 | ma3long2 | sportsman |
| 张译 | zhang1yi4 | actor | 易建联 | yi4jian4lian2 | sportsman |
| 段奕宏 | duan4yi4hong2 | actor | 刘翔 | liu2xiang2 | sportsman |
| 陈建斌 | chen2jian4bing1 | actor | 谌龙 | chen2long2 | sportsman |
| 李晨 | li2chen2 | actor | 孙杨 | sun1yang2 | sportsman |
| 马天宇 | ma3tian1yu3 | actor | 丁俊晖 | ding1jun4hui1 | sportsman |
| 胡歌 | hu2ge1 | actor | 金迪 | jin1di2 | actress |
| 吴镇宇 | wu2zhen4yu3 | actor | 王治郅 | wang2zhi4zhi4 | sportsman |
| 周星驰 | zhou1xing1chi3 | actor | 朱婷 | zhu1ting2 | sportsman |
| 任达华 | ren4da2hua2 | actor | 赵忠祥 | zhao4zhong1xiang2 | host |
| 古天乐 | gu3tian1le4 | actor | 倪萍 | ni2ping2 | host |
| 范冰冰 | fan4bing1bing1 | singer | 周立波 | zhou1li4bo1 | host |
| 李小龙 | li3xiao3long2 | actor | 谢娜 | xie4na4 | host |
| 徐熙娣 | xu2xi1di4 | host | 汪涵 | wang1han2 | host |
| 甄子丹 | zeng1zi3dan1 | actor | 王宁 | wang2ning2 | host |
| 冯远征 | feng2yuan3zheng1 | actor | 撒贝宁 | sa3bei4ning2 | host |
| 刘烨 | liu2ye4 | actor | 欧阳夏丹 | ou1yang2xia4dan1 | host |
| 汤唯 | tang1wei2 | actress | 李梓萌 | li3zi3meng2 | host |
| 丁海峰 | ding1hai3feng1 | actor | 柴静 | cai2jing4 | host |
| 潘玮柏 | pan1wei3bo2 | singer | 姜昆 | jiang1kun1 | actor |
| 周渝民 | zhou1yu2min2 | actor | 杨澜 | yang2lan2 | host |
| 黄秋生 | huang2qiu1sheng1 | actor | 李小萌 | li3xiao3meng2 | host |
| 王小利 | wang2xiao3li4 | actor | 水均益 | sui3jun1yi4 | host |
| 梁家仁 | liang2jia1ren2 | actor | 崔永元 | cui1yong3yuan2 | host |
| 赵本山 | zhao4ben3shan1 | actor | 邱孟煌 | qiu1meng4huang4 | host |
| 鹿晗 | lu4han2 | actor | 郎永淳 | duan4yong4chun4 | host |
| 王学兵 | wang2xue2bing1 | actor | 宋丹丹 | song4dan1dan1 | actress |
| 沙溢 | sha1yi4 | actor | 易中天 | yi4zhong1tian4 | scholar |
| 陈数 | chen2shu4 | actor | 朱时茂 | zhu1shi2mao4 | actor |
| 马英九 | ma3ying1jiu3 | politician | 高秀敏 | gao1xiu4min3 | actress |
| 张学友 | zhang1xue2you3 | singer | 郭冬临 | guo1dong1lin2 | actor |
|  |  |  | 潘长江 | pan1chang2jiang1 | actor |
