# Supplementary material for: Aging Effects on Phonological and Semantic Priming in the Tip-of-the-Tongue: Evidence From a Two-Step Approach
Source: Front Psychol. 2020 Feb 27;11:338. doi: 10.3389/fpsyg.2020.00338 (PMC7056892; doi:10.3389/fpsyg.2020.00338)
Supplement: Supplementary file 2 [file Data_Sheet_2.doc]

| Appendix B: Stimuli used in experiment 2. | | | | | | | |
| --- | --- | --- | --- | --- | --- | --- | --- |
| Target picture names | Pinyin | Occupation | Priming names | Pinyin | Occupations | Phonological relatedness (1 = first-name related; 2 = first-syllable related; 3 = unrelated) | Semantic relatedness (1 = related; 2 = unrelated) |
| 王健林 | wang2jian4lin2 | businessman | 王永庆 | wang2yong3qing3 | businessman | 1 | 1 |
| 刘强东 | liu2qiang2dong1 | businessman | 刘永好 | liu2yong3hao4 | businessman | 1 | 1 |
| 岳云鹏 | yu4yun2peng2 | actor | 岳跃利 | yue4yue4li4 | actor | 1 | 1 |
| 林保怡 | lin2bao3yi2 | actor | 林俊贤 | lin2jun4xian2 | actor | 1 | 1 |
| 黄忠泽 | huang2zhong1ze2 | actor | 黄志玮 | huang2zhi4wei3 | actor | 1 | 1 |
| 钟汉良 | zhong1han4liang2 | actor | 钟镇涛 | zhong1zhen4tao1 | actor | 1 | 1 |
| 蔡康永 | cai4kang1yong3 | actor | 蔡旻佑 | cai4min2you4 | actor | 1 | 1 |
| 佟丽娅 | tong2li4ya4 | actress | 佟晨洁 | tong2chen2jie2 | actress | 1 | 1 |
| 贾乃亮 | jia3nai3liang4 | actor | 贾延鹏 | jia3ya2peng2 | actor | 1 | 1 |
| 霍建华 | huo4jian4hua2 | actor | 霍亚明 | huo4ya4ming2 | actor | 1 | 1 |
| 梁朝伟 | liang2chao2wei3 | actor | 梁小龙 | liang2xiao3long3 | actor | 1 | 1 |
| 宋慧乔 | song4hui4qiao2 | actress | 宋翊菲 | song4yi4fei1 | actress | 1 | 1 |
| 宋小宝 | song4xiao3bao3 | actor | 宋俊龙 | song4jun4long2 | actor | 1 | 1 |
| 刘欢 | liu2hua1 | singer | 刘可 | liu2ke2 | singer | 1 | 1 |
| 李修平 | li3xiu1ping2 | host | 李瑞英 | li2rui4ying1 | host | 1 | 1 |
| 蔡明 | cai4ming2 | actress | 蔡琴 | cai4qin2 | actress | 1 | 1 |
| 赵丽蓉 | zhao4li4rong1 | actress | 赵咏华 | zhao4yong3hua2 | actress | 1 | 1 |
| 杨紫琼 | yang2zi3qiong2 | actress | 杨丽萍 | yang2li4ping2 | actress | 1 | 1 |
| 潘之琳 | pan1zhi1lin2 | actress | 潘迎紫 | pan1ying2zi3 | actress | 1 | 1 |
| 袁珊珊 | yuan2shan1shan1 | actress | 袁咏仪 | yuan2yong3yi2 | actress | 1 | 1 |
| 许亚军 | xu2ya4jun1 | actor | 徐浩峰 | xu2hao4feng1 | actor | 2 | 1 |
| 郑则仕 | zheng4ze2shi4 | actor | 甄志强 | zeng1zhi4qiang2 | actor | 2 | 1 |
| 姜大卫 | jiang1da4wei2 | actor | 江国宾 | wang1guo2bin1 | actor | 2 | 1 |
| 张子健 | zhang1zi3jian4 | actor | 章苏国 | zhang1su1guo2 | actor | 2 | 1 |
| 陈宝国 | chen2bao3guo2 | actor | 谌献波 | chen2xian4bo1 | actor | 2 | 1 |
| 王宝强 | wang2bao3qiang2 | actor | 汪坚辛 | wang1jian1xin1 | actor | 2 | 1 |
| 吴彦祖 | wu2yan4zu3 | actor | 伍伟乐 | wu3wei3le4 | actor | 2 | 1 |
| 蔡少芬 | cai4shao4fen1 | actress | 柴碧云 | cai2bi4yun2 | actress | 2 | 1 |
| 林志颖 | lin2zhi4ying3 | actor | 蔺达诺 | lin4da2ruo4 | actor | 2 | 1 |
| 高圆圆 | gao1yuan2yuan2 | actress | 郜妍妍 | gao4yan2yan2 | actress | 2 | 1 |
| 刘德华 | liu2de2hua2 | actor | 柳云龙 | liu3zhi4long2 | actor | 2 | 1 |
| 周杰伦 | zhou1jie2lun2 | singer | 邹志晨 | zou1zhi4chen2 | singer | 2 | 1 |
| 释小龙 | si4xiao3long3 | actor | 史震飞 | shi3zhen4fei1 | actor | 2 | 1 |
| 黎明 | li1ming2 | singer | 李解 | li3jie3 | singer | 2 | 1 |
| 陈坤 | chen2kun1 | actor | 谌伟 | chen2wei2 | actor | 2 | 1 |
| 高雄 | gao1xiong2 | actor | 郜乾 | gao4qian2 | actor | 2 | 1 |
| 王祖蓝 | wang2zu3lan2 | actor | 汪东城 | wang1dong1cheng2 | actor | 2 | 1 |
| 刘恺威 | liu2kai3wei1 | actor | 柳小海 | liu2xiao3hai3 | actor | 2 | 1 |
| 殷桃 | yin1tao2 | actress | 尹媗 | yin2xuan1 | actress | 2 | 1 |
| 白百合 | bai2bai3he2 | actress | 柏嘉莹 | bai3jia1ying2 | actress | 2 | 1 |
| 朱茵 | zhu1yin1 | actress | 刘芸 | liu2yun2 | actress | 3 | 1 |
| 于震 | yu2zhen4 | actor | 罗文 | luo2wen2 | actor | 3 | 1 |
| 焦恩俊 | jiao1en1jun4 | actor | 黄日华 | huang2ri4hua2 | actor | 3 | 1 |
| 王志飞 | wang2zhi4fei1 | actor | 张颂文 | zhang1song4wen2 | actor | 3 | 1 |
| 侯勇 | hou2yong3 | actor | 汪粤 | wang1yue4 | actor | 3 | 1 |
| 张雨生 | zhang1yu2sheng1 | singer | 高凌风 | gao1ling2feng1 | singer | 3 | 1 |
| 吴克群 | wu2ke4qun2 | singer | 唐禹哲 | tang2yu2zhe2 | singer | 3 | 1 |
| 方大同 | fang1da4tong2 | singer | 余铭轩 | yu2ming2xuan1 | singer | 3 | 1 |
| 任贤齐 | ren4xian2qi2 | singer | 李佳明 | li3jia1ming3 | singer | 3 | 1 |
| 梁咏琪 | liang2yong3qi2 | singer | 陈妍希 | chen1yan2xi1 | singer | 3 | 1 |
| 容祖儿 | rong2zu3er2 | singer | 何韵诗 | he2yun4shi1 | singer | 3 | 1 |
| 关晓彤 | guan1xiao3tong2 | actress | 陈妙瑛 | chen2miao4ying1 | actress | 3 | 1 |
| 李永波 | li3yong3bo1 | sportsman | 蒋梦麟 | jiang3meng4lin2 | sportsman | 3 | 1 |
| 康辉 | kang1hui1 | host | 张政 | zhang1zheng4 | host | 3 | 1 |
| 海霞 | hai3xia2 | host | 文清 | wen2qing1 | host | 3 | 1 |
| 白岩松 | bai2yan2song1 | host | 崔志刚 | cui1zhi4gang1 | host | 3 | 1 |
| 张泽群 | zhang1ze2qun2 | host | 李咏 | li2yong3 | host | 3 | 1 |
| 黄宏 | huang2hong2 | actor | 马季 | ma3ji4 | actor | 3 | 1 |
| 彭于晏 | peng2yu2yan4 | actor | 胡宇威 | hu2yu3wei1 | actor | 3 | 1 |
| 胡军 | hu2jun1 | actor | 陈晓 | chen2xiao3 | actor | 3 | 1 |
| 吴奇隆 | wu2qi2long2 | actor | 伍思凯 | wu3si1kai3 | singer | 1 | 2 |
| 张国强 | zhang1guo2qiang2 | actor | 张国焘 | zhang1guo2tao1 | politician | 1 | 2 |
| 魏坤琳 | wei4kun2lin2 | scholar | 魏炳桦 | wei4bing3ye4 | actor | 1 | 2 |
| 黄晓明 | huang2xiao3ming2 | actor | 黄家驹 | huang2jia1ju1 | singer | 1 | 2 |
| 梁家辉 | liang2jia1hui1 | actor | 梁晓声 | liang2xiao3sheng1 | writer | 1 | 2 |
| 马伊琍 | ma3yi1li4 | actress | 马小艺 | ma3xiao3yi4 | host | 1 | 2 |
| 孙楠 | sun1nan2 | singer | 孙权 | sun1quan2 | politician | 1 | 2 |
| 邓丽君 | deng4li4jun4 | singer | 邓颖超 | deng4ying3chao1 | writer | 1 | 2 |
| 罗大佑 | luo2da4you4 | actor | 罗瑞卿 | luo2rui4qing1 | politician | 1 | 2 |
| 杨坤 | yang2kun2 | singer | 杨威 | yang2wei1 | sportsman | 1 | 2 |
| 汪峰 | wang1feng1 | singer | 汪洋 | wang1yang2 | politician | 1 | 2 |
| 吴孟达 | wu2meng4da2 | actor | 吴大维 | wu2da4xiong2 | singer | 1 | 2 |
| 刘国梁 | liu2guo2liang2 | sportsman | 刘天佐 | liu2tian1zuo3 | scholar | 1 | 2 |
| 陈羽凡 | chen1yu2fan2 | actor | 陈独秀 | chen2du2xiu4 | politician | 1 | 2 |
| 金星 | jin1xing1 | host | 金莎 | jin1sha1 | singer | 1 | 2 |
| 郭德纲 | guo1de2gang1 | wen | 郭艾伦 | guo1ai4lun2 | sportsman | 1 | 2 |
| 郭达 | guo1da | actor | 郭峰 | guo1feng1 | singer | 1 | 2 |
| 范伟 | fan4wei3 | actor | 樊凡 | fan2fan2 | singer | 1 | 2 |
| 孔令辉 | kong3ling4hui1 | sportsman | 孔庆三 | kong3qing4san1 | actor | 1 | 2 |
| 董明珠 | dong3ming2zhu1 | businessman | 董圆圆 | dong3yuan2yuan2 | actress | 1 | 2 |
| 杨怡 | yang2yi2 | actress | 央金 | yang1jin1 | singer | 2 | 2 |
| 苏有朋 | su1you3peng2 | actor | 粟裕 | su4yu4 | politician | 2 | 2 |
| 陈百祥 | chen2bai3qiang2 | actor | 谌贻琴 | chen2yi2qin2 | politician | 2 | 2 |
| 刘小峰 | liu2shao4feng1 | actor | 柳宗元 | liu3zhong1yuan2 | writer | 2 | 2 |
| 王丽坤 | wang2li4kun4 | actress | 汪佩蓉 | wang1pei4rong2 | singer | 2 | 2 |
| 吴秀波 | wu2xiu4bo1 | actor | 伍洲彤 | wu3zhou1tong2 | host | 2 | 2 |
| 李易峰 | li2yi4feng1 | actor | 栗战书 | li4zhan4shu1 | politician | 2 | 2 |
| 何润东 | he2run4dong1 | actor | 贺国强 | he4guo2qiang2 | politician | 2 | 2 |
| 关婷娜 | guan1ting2na4 | actress | 管文蔚 | guan3wen2wei4 | politician | 2 | 2 |
| 关悦 | guan1yue4 | actress | 管彤 | guan3tong2 | host | 2 | 2 |
| 林丹 | lin2dan1 | sportsman | 蔺欣 | lin4xin2 | writer | 2 | 2 |
| 乐嘉 | le4jia1 | host | 乐桐 | le4tong2 | singer | 2 | 2 |
| 张艺谋 | zhang1yi4mou2 | director | 章均赛 | zhang1jun1sai4 | politician | 2 | 2 |
| 孟非 | meng4fei1 | host | 蒙毅 | meng2yi4 | politician | 2 | 2 |
| 朱军 | zhu1jun1 | host | 祝川 | zhu4chuan1 | actor | 2 | 2 |
| 敬一丹 | jing4yi1dan1 | host | 金玉婷 | jin1yu4ting2 | actress | 2 | 2 |
| 张绍刚 | zhang1shao4gang1 | host | 章绍伟 | zhang1shao4wei3 | actor | 2 | 2 |
| 陈佩斯 | chen2pei4si1 | actor | 谌俊军 | chen2jun4jun1 | businessman | 2 | 2 |
| 罗志祥 | luo2zhi4xiang2 | singer | 洛葳 | luo4wei1 | actor | 2 | 2 |
| 蔡国庆 | cai4guo2qing4 | singer | 柴京云 | cai2jing1yun2 | actor | 2 | 2 |
| 陈寒柏 | chen2han2bai3 | actor | 郭沫若 | guo1mo4luo4 | writer | 3 | 2 |
| 俞敏洪 | yu3min3hong2 | businessman | 王家卫 | wang2jia1wei4 | director | 3 | 2 |
| 杜淳 | du4chun2 | actor | 于谦 | yu2qian1 | politician | 3 | 2 |
| 谢贤 | xie4xian2 | actor | 闫肃 | yan2su4 | writer | 3 | 2 |
| 于荣光 | yu2rong2guang1 | actor | 申方剑 | shen1fang1jian4 | host | 3 | 2 |
| 赵又廷 | zhao4you4ting2 | actor | 钟南山 | zhong1nan2shan1 | scholar | 3 | 2 |
| 张国强 | zhang1guo2qiang2 | actor | 吕叔湘 | lv3shu1xiang1 | scholar | 3 | 2 |
| 唐国强 | tang2guo2qaing2 | actor | 龚伟杰 | gong1wei3jie2 | sportsman | 3 | 2 |
| 朱镕基 | zhu1rong2ji1 | politician | 李茂山 | li2mao4shan1 | singer | 3 | 2 |
| 刘若英 | liu2ruo4ying1 | singer | 郭霄珍 | guo1xiao1zhen1 | actress | 3 | 2 |
| 胡彦斌 | hu2yan4bing1 | singer | 梁思成 | liang2si1cheng2 | scholar | 3 | 2 |
| 王楠 | wang2nan2 | sportsman | 陈琳 | chen2lin2 | singer | 3 | 2 |
| 秦凯 | qin2kai3 | sportsman | 赵雷 | zhao4lei2 | singer | 3 | 2 |
| 吴敏霞 | wu2min3xia2 | sportsman | 郭霄珍 | guo1xiao1zhen | actor | 3 | 2 |
| 邹市明 | zou4shi4ming2 | sportsman | 王小波 | wang2xiao3bo1 | writer | 3 | 2 |
| 郎平 | lang2ping2 | sportsman | 吕中 | lv3zhong1 | actress | 3 | 2 |
| 何炅 | he2jiong2 | host | 郭军 | guo1jun1 | actor | 3 | 2 |
| 朱迅 | zhu1xun4 | host | 高亮 | gao1liang4 | actor | 3 | 2 |
| 廖凡 | liao4fan2 | actor | 冷漠 | leng3mo4 | singer | 3 | 2 |
| 井柏然 | jing4bo2ran2 | singer | 陈荣竣 | chen2rong2jun4 | actor | 3 | 2 |
